# Flourishing at work: Nurses' motivation through daily communication – An ethnographic approach

**DOI:** 10.1111/nhs.12789

**Published:** 2020-11-16

**Authors:** Carina Ahlstedt, Carin Eriksson Lindvall, Inger K. Holmström, Åsa Muntlin

**Affiliations:** ^1^ Department of Public Health and Caring Sciences, Health Services Research Uppsala University Uppsala Sweden; ^2^ Department of Business Studies Uppsala University Uppsala Sweden; ^3^ School of Health, Care and Social Welfare Mälardalen University Västerås Sweden; ^4^ Department of Medical Sciences Uppsala University Uppsala Sweden; ^5^ Department of Emergency Care and Internal Medicine Uppsala University Hospital Uppsala Sweden

**Keywords:** communication, healthcare sector, motivation, nurses, self‐determination theory, workplace interdisciplinary communication

## Abstract

Shortage and turnover of registered nurses are worldwide challenges, and work motivation is one factor in retaining staff in the healthcare sector. The aim of this study was to explore registered nurses' motivation expressed in daily communication, using the basic needs in self‐determination theory as a framework. A secondary analysis of ethnographic data, collected through participant observations, informal interviews during observations, and individual interviews, was used. A total sample of all registered nurses employed at a hospital unit in Sweden (*n* = 10) participated. The data were analyzed thematically through the lens of the basic needs in self‐determination theory: *autonomy*, *competence*, and *relatedness*. Self‐regulation of learning, the possibilities to discuss work‐related challenges with colleagues, and having registered nurses lead dialogues with physicians were factors connected to *autonomy*. Having a registered nurse and physician solve problems together was a factor connected to *competence*. A sense of belonging and security in a permissive climate between registered nurses was connected to *relatedness*. This paper has implications for increased awareness of the three basic motivational needs, which could be used in the development of attractive workplaces.

## 1 INTRODUCTION

Shortage and turnover of registered nurses in healthcare are worldwide challenges (Hayes et al., 2012), associated with patient safety risks and higher patient mortality (Ball et al., 2018). The World Health Organization highlighted work motivation as one of the factors important for resolving problems with recruiting and retaining staff in the healthcare sector (World Health Organization, 2016). A definition of motivation at work is a drive to do something, focused on what energizes and gives direction in performing work tasks (Ryan & Deci, 2017). In this study, we have focused on registered nurses' motivation expressed in daily communication, since communication between registered nurse colleagues and with other professions is fundamental in the care of patients (Robb, Barrett, Komaromy, & Rogers, 2004; Boynton, 2015; Edmondson, 2018).

## 2 BACKGROUND

### 2.1 Work motivation

A number of factors influence registered nurses' motivation at work. For example, experiencing that the work is meaningful is crucial for motivation (Perreira, Innis, & Berta, 2016; Toode, Routasalo, & Suominen, 2011). The opportunity to learn and develop in daily work, preferably together with colleagues in the same profession, is a vital factor for motivation (Ahlstedt, Eriksson Lindvall, Holmström, & Muntlin Athlin, 2019; Toode et al., 2011). Further, having good relationships with colleagues, while being able to work independently, is important (Ahlstedt et al., 2019; Toode et al., 2011). There is a need for a deeper understanding of registered nurses' work motivation expressed in daily communication with colleagues, since communication is fundamental to their work. Therefore, we used self‐determination theory to study the registered nurses' communication with colleagues, in order to further expand knowledge of their motivation in daily work.

### 2.2 Self‐determination theory and motivation at work

**FIGURE 1:**
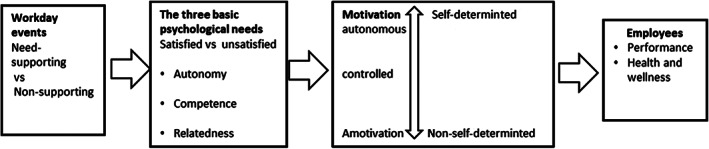
A modified illustration of the self‐determination theory (based on Ryan and Deci, 2017)

One well‐known motivation theory is self‐determination theory (Ryan & Deci, 2017). Previous studies from various cultures and settings around the world have used the basic needs in self‐determination theory to better understand work‐related motivation (Ryan & Deci, 2017; Van den Broeck, Ferris, Chang, & Rosen, 2016). Self‐determination theory is a macro theory and describes a continuum between controlled motivation and autonomous motivation (Ryan & Deci, 2000, 2017). The definition of controlled motivation is when employees work because of external regulation, with low levels of self‐determination. Autonomous motivation arises when employees work because of enjoyable work tasks or self‐selected goals, with high levels of self‐determination (Ryan & Deci, 2017). According to self‐determination theory, humans have three basic needs that have an impact on motivation (Ryan & Deci, 2017). The first is the need for a perception of *autonomy*, which relates to a feeling of one's own willingness and ownership of one's actions, being self‐regulating in different situations. The second is the need to feel *competent*, which refers to the experiencing of opportunities and support for expansion and expression of our capacity and talents, to operate effectively and master the situation. The third is the need for *relatedness*, the feeling of being connected with others and having a sense of belonging (Gagné & Deci, 2005; Ryan & Deci, 2017), see Figure 1.

Self‐determination theory emphasizes that workplaces where employees feel supported in their autonomous motivation will lead to increased employee satisfaction, performance, and organizational efficiency. In other words, the work will be a platform where the employees can flourish (Deci, Olafsen, & Ryan, 2017). Furthermore, employees experience less exhaustion, burnout, and ill health when they are autonomously motivated (Deci et al., 2017; Ryan & Deci, 2017).

However, as far as we know, self‐determination theory has not previously been applied to registered nurses' daily communication. Knowledge of the basic motivational needs and the perception of self‐determination may support an organizational culture where registered nurses want to work, and hence may provide one piece in solving the puzzle of nurse shortage and turnover. The aim of this study was therefore to explore registered nurses' motivation expressed in daily communication, using the basic needs in self‐determination theory as a framework.

## 3 METHODS

We undertook a secondary analysis of ethnographic data, collected through participant observations, informal interviews during observations, and individual interviews. The analysis explored registered nurses' work motivation expressed in daily communication and reflected in self‐determination theory. The use of existing data can be valuable, since participants' time and effort in a study become more worthwhile (Heaton, 2008; Ruggiano & Perry, 2019). The inner work life theory (Amabile & Kramer, 2011) was used in the original study to explore registered nurses' motivation during workday events (Ahlstedt et al., 2019).

### 3.1 Sample and setting

The setting was a unit linked to an emergency department at a university hospital in Sweden. Registered nurses worked with different colleagues in three shifts: morning, afternoon, or night. The physicians who were on call changed every week. A total sample of all registered nurses employed at the unit (*n* = 10), two males and eight females, participated. The participants' average age was 32 years (range 23–57 years). The sample and setting are described in detail in the original study (Ahlstedt et al., 2019).

### 3.2 Data collection

Data from participant observations and informal interviews during observations were collected over the course of 4 months in 2016 (total 56 h). A total of 479 events and 58 informal interviews during observations were documented in field notes. This method provides insights into the unspoken elements and connects the researcher more closely to the basic human experience (Patton, 2015). Additional individual interviews (*n* = 9) were conducted soon after the final observation period, to give the registered nurses more time to express what affected their personal motivation in daily work. Data collection was conducted by the first author. The author had no connection to the unit before the study started. The data collection is described in detail in the original study (Ahlstedt et al., 2019).

### 3.3 Data analysis

A reflective thematic approach was used (Braun & Clarke, 2006). This method, involving a six‐phase analytic process, offers flexibility and a possibility for theoretical interpretation (Terry, Hayfield, Clarke, & Braun, 2017). In phase 5, the sub‐themes were analyzed in relation to the three theoretical themes, that is, the three basic needs according to self‐determination theory: autonomy, competence, and relatedness (Deci et al., 2017). The analysis process moved back and forth between the phases, specifically between phases 4 and 5.

1. The first phase involved two parallel processes, one involving familiarization through reading all the data in the original dataset and the other involving selective coding, identifying registered nurses' motivation in communication situations. Communication was in focus when registered nurses expressed that something in the communication gave them energy in a positive direction, based on the definition of motivation (Ryan & Deci, 2017). The data from the selective coding included communication between registered nurses and communication between registered nurses and physicians, which provided the basis for two overarching themes.
2. The second phase involved labelling all segments of relevance within the two overarching themes and generation of codes.
3. The third phase involved searching for patterns, clustering codes, and beginning the construction of the sub‐themes.
4. In the fourth phase, these sub‐themes were reviewed to ensure that they worked well in relation to the cluster of codes, the data, and the full dataset.
5. In the fifth phase, we analyzed the sub‐themes in relation to the three theoretical themes, that is, the three basic needs according to self‐determination theory – autonomy, competence, and relatedness (Ryan & Deci, 2017) – within each overarching theme.
6. The final phase involved producing the report.

The software program Nvivo 12 was used to manage the empirical data, and the first author performed the main analysis. To ensure analysis consistency, the three co‐authors read the dataset, the data corpus, and repeatedly discussed the coding and categorization.

### 3.4 Ethical considerations

The project was approved by the Regional Ethical Review Board in Sweden (Dnr 2015/491) and followed the guidelines of the Helsinki Declaration (World Medical Association, 2013). The participants received verbal and written information, and gave written informed consent, and all registered nurses were given fictitious names in the results. Data were processed and stored ensuring confidentiality. No patients were observed.

## 4 RESULTS

Two overarching themes were established: *Communication between registered nurses* and *Communication between registered nurse and physician*. The findings within each overarching theme are related to the basic motivational needs in self‐determination theory: *autonomy*, *competence*, and *relatedness* (Tables 1 and 2).

**TABLE 1:** Registered nurses' work motivation in daily communication between registered nurses, framed using the basic needs in self‐determination theory: *autonomy, competence, and relatedness*

Overarching theme: Communication between registered nurses		
Themes: Basic needs	Sub‐themes	Cluster codes
The perspective of autonomy	*Self‐regulation of own learning together with other registered nurses*	Extending own knowledge by asking questions. Learning as a result of solving tasks with registered nurse colleagues.
	*The opportunity for independence based on sufficient information*	The registered nurses support each other with different pieces of information, which together create the full picture needed by a registered nurse to make autonomous decisions in daily work. The registered nurse receives structured handovers, creating an opportunity for independence.
The perspective of competence	*Enough competence to support other registered nurses and ask relevant questions*	Asking and answering each other's relevant questions during daily work. The registered nurses give structured and correct information to one another during daily work when necessary.
The perspective of relatedness	*A permissive climate between the registered nurses*	A sense of support and security in relation to other registered nurses. Asking each other questions with respect, listening to the answers, and acknowledging each other. Ongoing communication and a few minutes of social small talk between registered nurses, resulting in a sense of belonging.

**TABLE 2:** Registered nurses' work motivation in daily communication between registered nurse and physician, framed using the basic needs in self‐determination theory: *autonomy*, *competence* and *relatedness*

Overarching theme: Communication between registered nurse and physician		
Themes: Basic needs	Sub‐themes	Cluster codes
The perspective of autonomy	*Leads the dialogues with self‐confidence*	The registered nurse leads the dialogue, asks questions, and clarifies what will be done, taking the role of a team leader. The physician trusts the registered nurse's knowledge.
The perspective of competence	*The registered nurse's knowledge is visible*	The physician asks factual questions, and the registered nurse can answer immediately. The physician and the registered nurse solve problems together, with questions and answers in both directions.
The perspective of relatedness	*The physician sees the person beyond the registered nurse's professional role by engaging in social small talk*	Good atmosphere between the registered nurse and the physician. The registered nurse feels that the physician has an interest in seeing the person beyond the professional role.
	*An understanding and a sense of equality and respect*	The registered nurse feels that the physician listens to him/her. A sense of respect between the different professional roles. The registered nurse is confident that the physician and the registered nurse understand each other. Asking questions with a sense of security.

### 4.1 Communication between registered nurses

The perspective of *autonomy* had two sub‐themes. The first sub‐theme, *Self‐regulation of own learning together with other registered nurses*, refers to when registered nurses extended their own knowledge because they desired to do so. This involved, for instance, when a registered nurse learned through asking colleagues and in situations where registered nurses solved tasks together during daily communication.

“Yes, developing is the most important thing. We work together in caring for all patients and helping each other. Then you learn from one another and it is more fun to work.” (Interview during Observation no. 36)

The second sub‐theme, *The opportunity for independence based on sufficient information*, describes situations when registered nurses were satisfied because they received information needed to make autonomous decisions during daily work. This could be when a registered nurse received information that was necessary for planning their own daily work from a colleague during daily communication or in a well‐structured handover. Some of the registered nurses used an SBAR (situation, background, assessment, and recommendation) structure in handovers, which gave a clear overview of a patient's situation and what should be done in the next step.

Lee's description of getting a report in an SBAR structure: “I feel a little harmonious in my body, [laughter] because then I know what to do … then, I become calm and stable as well. You feel quite confident in your professional role, in addition to getting this safety. Okay, I have full control of the situation; then something can always happen, but I have full control of the situation as it is right now.” (Individual Interview no. 8)

The perspective of *competence* was reflected in the sub‐theme *Enough competence to support other registered nurses and ask relevant questions*, which describes situations when a registered nurse felt skilled enough to provide support to other registered nurses, when needed, through communication. In addition, when registered nurses could ask and get answers to relevant questions, this could strengthen their sense of competence.

Daryl is standing next to the sample collection cart. She looks closely at one test tube. It seems as if she is thinking deeply about something. After a while, Daryl asks Gail how the tube works. Gail describes the specific tube's function and gives examples when needed. (Event no. 179)

The sub‐theme under the perspective of *relatedness, A permissive climate between the registered nurses*, refers to when the communication atmosphere was free from prestige. For instance, registered nurses asked questions with respect, listened to the answers, and acknowledged each other. Registered nurses also felt togetherness and security when engaging in brief small talk with other registered nurses during their daily work.

Julia and Alex help each other sort out uncertainties using the prescription, the care manual, and the daily activity whiteboard. Both are equally involved in the dialogue. It is a permissive atmosphere, where they are helping each other in a developing way. (Event no. 174)

“It feels great because they [the registered nurse colleagues] are all so nice and you can be honest, ‘I feel uncertain about this’ and then they say ‘So am I, but let's figure this out together.’ Or ‘You seem to know this, can I ask you a question?’.” (Individual Interview no. 2)

Marion and Noel have read up on the patient's pain and the analgesic medication that the patient has been given and that can be administered if needed. The registered nurses agree on what they should give, based on the “on‐demand medications list.” It seems as if there is a sense of security between the nurses in their dialogue. (Event no. 453)

### 4.2 Communication between registered nurse and physician

The perspective of *autonomy* could be seen in the sub‐theme *Leads the dialogues with self‐confidence*, and involves, for example, when registered nurses led dialogues during rounds, asked questions, or clarified what would be done in a summary. This gave them a clearer picture of the situation and helped them know what to do in the next step and create their own plan. It was considered essential that the physicians trusted the registered nurses' knowledge.

Lee leads the dialogue and asks, for instance “What should the blood pressure be for this patient? What is the goal?” Lee summarizes the most important points for further planning of the work. (Event no. 46)

Shannon receives information about a new patient from a physician. She asks about diagnosis, controls, and – when needed – asks for clarification regarding what she has read about the patient. Shannon is clear on what the physician needs to do before they can admit the patient. (Event no. 37)

The perspective of *competence* was reflected in the sub‐theme *The registered nurse*'*s knowledge is visible*. This refers to when a registered nurse could immediately answer a physician's questions, thus creating a sense of competence. Furthermore, it was important that the physician and the registered nurse solved problems together, with questions and answers coming from both parties.

Two physicians discuss patient cases. Kelly works with her duties, but listens to the physicians and answers their questions quickly. (Event no. 23)

“It is nice when the physician knows what has happened. I can then add the information I have about events, for example what has happened during the evening. And then the physician says ‘Let's do this and this…’ and then I say ‘We should also do this,’ and that is the dialogue.” (Individual Interview no. 9)

The perspective of *relatedness* had two sub‐themes. The first, *The physician sees the person beyond the registered nurse*'*s professional role by engaging in social small talk*, describes the importance of a good atmosphere between a registered nurse and a physician. For instance, the registered nurse felt that the physician had an interest in the person behind the professional role when there were some brief moments of small talk during their daily work.

“Yes, that is, you feel like you get in touch with the person. So it is the most fun to work when you can talk seriously, but also talk about other things. And that you feel that you dare to ask questions, and you get a good response and the person has an interest in talking to you.” (Individual Interview no. 1)

The next sub‐theme, *An understanding and a sense of equality and respect*, refers to when a registered nurse felt that a physician listened, and the registered nurse and the physician had a mutual understanding of each other in the communication. This occurred when a registered nurse felt a sense of security, dared to ask a physician questions, and was not ignored. Another example was when a physician invited a registered nurse to ask if there was something that was unclear in the communication. Furthermore, seen from a perspective of well‐functioning relatedness, there was a sense of equality and respect between the registered nurse and the physician in their communication.

Ashley gives a suggestion on how to handle a situation with a patient. The physician says that it is a good proposal. The communication between Ashley and the physician seems good. Each listens to the other. Ashley looks very happy. (Event no. 110)

A physician and Sidney discuss a patient's pain treatment. The discussion results in a shared strategy. (Event no. 130)

## 5 DISCUSSION

### 5.1 Basic motivational needs

Our findings encompass registered nurses' three basic motivational needs – *autonomy, competence*, and *relatedness* – in daily communication, in accordance with self‐determination theory. A deeper knowledge of motivation in daily work could support an organizational culture where registered nurses would want to continue to work. We emphasize that there are benefits in highlighting the different motivational needs separately and specifically, even though they are interconnected and interact, as this enables the creation of tools to improve motivation through communication during daily work. Therefore, in this section, we focus on each of these perspectives in turn.

#### 5.1.1 The autonomy perspective

The findings highlighted registered nurses' self‐regulation of learning in daily communication with other registered nurses. For instance, solving problems together was important for autonomy. This is in line with previous research of other professions, which has found a strong relationship between one's own opportunity to learn and self‐regulation according to self‐determination theory (e.g., Black & Deci, 2000; Levesque, Zuehlke, Stanek, & Ryan, 2004). Therefore, it is important to organize work so that it creates opportunities for registered nurses to reflect and discuss work‐related challenges with one another during their daily work. Another finding that was essential for autonomy was when a registered nurse received well‐organized information during handovers, since this made it possible to plan and take control of their own daily work. In addition, it is well known that a clear and sufficient handover is important from a patient safety perspective (De Meester, Verspuy, Monsieurs, & Van Bogaert, 2013). This knowledge is valuable for organizations, to ensure that handover situations work well from both these perspectives.

Regarding the need for autonomy during communication with physicians, it was important for the registered nurse to take the lead in dialogues with self‐confidence. An example of a communication situation was when the registered nurse held ward rounds, asked questions when needed, and summarized what would be done. In addition, ward rounds held by registered nurses can have a positive effect on patient outcomes, such as infection control and hospital‐acquired pressure ulcers (Fisher, Grosh, & Felty, 2016; Garvey et al., 2019). Therefore, it can be important for organizations to implement nurse‐led ward rounds from both the work motivation perspective and the patient safety perspective.

The autonomy perspective focuses on different ways to experience independence during work, while knowledge is the focus of the competence perspective (Ryan & Deci, 2017). This is described in the next section.

#### 5.1.2 The competence perspective

In communication with a registered nurse colleague, the basic need of competence was expressed when the registered nurses had enough knowledge to ask relevant questions. In addition, situations when a registered nurse gave structured and relevant information to colleagues during daily work can be seen as a consequence of feeling confident with one's own knowledge and with collegial support. This is also related to the need of competence in self‐determination theory (Ryan & Deci, 2017). In line with this, previous research has indicated collegial support as one factor that may help reduce turnover among staff (Noguchi‐Watanabe, Yamamoto‐Mitani, & Takai, 2016). Organizations may gain from creating the conditions for registered nurses to learn and develop their own knowledge together with colleagues in their daily work.

The perspective of competence was also relevant when a registered nurse's own knowledge was visible during communication with a physician. The registered nurse and physician solved problems together, with questions and answers from both directions. Engaging in communication with physicians to identify solutions to problems together and participating in decision‐making are among the professional skills of nurses (Apker, Propp, Zabava Ford, & Hofmeister, 2006).

Collaboration requires well‐functioning relationships with others. The third basic motivational need in self‐determination theory, relatedness, focuses more specifically on relations with others (Ryan & Deci, 2017) and will be discussed in the next section.

#### 5.1.3 The relatedness perspective

The importance of relatedness, as described in self‐determination theory (Ryan & Deci, 2017), in communication between registered nurses, was seen when a nurse felt a sense of belonging and security in a permissive climate. Similar findings have been identified in other research, linking positive collegial relationships to job satisfaction (Ylitörmänen, Turunen, Mikkonen, & Kvist, 2019) and work performance (Amabile & Kramer, 2011). Our findings, together with previous research (Ylitörmänen et al., 2019), emphasize the importance of organizations creating the possibility for registered nurses to communicate in a way that supports intra‐professional collegial relationships in daily work.

Another notable finding was the sense of relatedness and motivation when a registered nurse and a physician saw one another beyond their professional roles, by engaging in small talk. It is well‐known in organizational research that small talk has a key role in building trust and relationships between employees. Small talk builds social cohesiveness and is important for organizations' effectiveness (Coupland, 2003; Keyton et al., 2013). In addition, registered nurses' perceptions of physicians' communication, humor, empathy, clarity, immediacy, and listening are important for collaboration and registered nurses' job satisfaction (Wanzer, Wojtaszczyk, & Kelly, 2009). Therefore, it is crucial to ensure that there is an organizational culture where physicians and registered nurses can engage in small talk, as they need to collaborate in different ways during their daily work.

Our findings highlight that understanding and a sense of equality, respect, and security between a registered nurse and a physician appear to be central in communication for the registered nurse's feeling of relatedness, as described in self‐determination theory (Ryan & Deci, 2017). This is important since – according to previous research – registered nurses' and physicians' well‐functioning collaboration and communication could promote a work environment that reduces registered nurses' intentions to leave their profession (Al‐Hamdan, Banerjee, & Manojlovich, 2018; Galletta, Portoghese, Carta, D'Aloja, & Campagna, 2016; Sasso et al., 2019). In addition, well‐functioning teamwork and a good collegial relationship between a registered nurse and a physician could increase patient safety (Smeds Alenius, Tishelman, Runesdotter, & Lindqvist, 2014). Moreover, it is well‐known that serious healthcare mistakes have been reduced in health organizations where employees feel comfortable in providing support to one another, asking questions, and speaking out about their mistakes (Edmondson, 2018). Barriers to effective teamwork include a hierarchical structure between different professionals, and when professional groups have differing expectations on how work should be done (Weller, Boyd, & Cumin, 2014).

### 5.2 Strengths, limitations, and future research

In relation to trustworthiness, one limitation is the reliance on secondary analysis, since the data were originally collected for another purpose. Two strengths are the relatively short time between the data collection and the secondary analysis and having the same four researchers for both the data collection and this study (Ruggiano & Perry, 2019). Further, to achieve dependability, all four authors were involved in the analysis, which contributed to ensuring that the process was logical and documented. A strength in relation to credibility was the ethnographic approach with data triangulation. Further, the observations provided the researcher a possibility to get closer to actual events, getting so‐called first‐hand experience (Patton, 2015). To achieve confirmability, quotes are presented that reflect the participants' voices. In relation to transferability, a limitation was that the data collection was carried out at only one unit at a hospital in central Sweden. A strength, however, was the total sample of all registered nurses at the unit. Furthermore, the reflections on the findings in relation to the universal self‐determination theory strengthen the possibility of the findings being transferable.

This study was from the registered nurse perspective. It would be interesting to use self‐determination theory in future research of other health professionals' perspectives. In further studies, it could also be of interest to deepen the knowledge of daily motivation from the perspective of psychological safety in accordance with Edmondson (2018). This could provide more comprehensive knowledge about how to further develop an attractive workplace in the healthcare organization.

## 6 CONCLUSION AND CLINICAL IMPLICATIONS

The findings shed light on the possibilities to promote self‐regulated motivation in registered nurses' daily communication. Managers and leaders are important for creating good communication. They may need to pay attention to the relationships between registered nurses and physicians in communication situations, to avoid inter‐professional barriers and strengthen motivation and relatedness. Promoting small talk and a sense of equality, respect, and security between registered nurses and physicians appears to be central. Furthermore, our findings emphasize the importance of creating the possibility for registered nurses to communicate with each other, in a way that supports the intra‐professional collegial relationship and strengthens motivation in daily work. Lastly, this study may contribute to increased awareness of the three basic motivational needs – *autonomy*, *competence*, and *relatedness* – and be helpful for creating work environments where registered nurses want to work and can flourish.

## CONFLICT OF INTEREST

The authors declare they have no conflicts of interests.

## AUTHOR CONTRIBUTIONS

Study design: C.A., I.K.H., C.E.L., and Å.M.

Data collection: C.A.

Data analysis: C.A., I.K.H., C.E.L., and Å.M.

Manuscript writing: C.A.

Supervision and feedback on manuscript: I.K.H., C.E.L., and Å.M.

## References

[nhs12789-bib-0001] Ahlstedt, C. , Eriksson Lindvall, C. , Holmström, I. K. , & Muntlin Athlin, Å. (2019). What makes registered nurses remain in work? An ethnographic study. International Journal of Nursing Studies, 89, 32–38. 30339953 10.1016/j.ijnurstu.2018.09.008

[nhs12789-bib-0002] Al‐Hamdan, Z. , Banerjee, T. , & Manojlovich, M. (2018). Communication with physicians as a mediator in the relationship between the nursing work environment and select nurse outcomes in Jordan. Journal of Nursing Scholarship, 50(6), 714–721. 30043513 10.1111/jnu.12417

[nhs12789-bib-0003] Amabile, T. , & Kramer, S. (2011). The progress principle: Using small wins to ignite joy, engagement, and creativity at work. Boston, MA: Harvard Business Review Press.

[nhs12789-bib-0004] Apker, J. , Propp, K. M. , Zabava Ford, W. S. , & Hofmeister, N. (2006). Collaboration, credibility, compassion, and coordination: Professional nurse communication skill sets in health care team interactions. Journal of Professional Nursing, 22(3), 180–189. 16759961 10.1016/j.profnurs.2006.03.002

[nhs12789-bib-0005] Ball, J. E. , Bruyneel, L. , Aiken, L. H. , Sermeus, W. , Sloane, D. M. , Rafferty, A. M. , … RN4Cast Consortium . (2018). Post‐operative mortality, missed care and nurse staffing in nine countries: A cross‐sectional study. International Journal of Nursing Studies, 78, 10–15. 28844649 10.1016/j.ijnurstu.2017.08.004 PMC5826775

[nhs12789-bib-0006] Barrett S., Robb M., Komaromy C., & Rogers A. (Eds.). (2004). Communication, relationships and care: A reader. London, UK: Routledge.

[nhs12789-bib-0007] Black, A. E. , & Deci, E. L. (2000). The effects of instructors' autonomy support and students' autonomous motivation on learning organic chemistry: A self‐determination theory perspective. Science Education, 84(6), 740–756.

[nhs12789-bib-0008] Boynton, B. (2015). Successful nurse communication: Safe care, healthy workplace, & rewarding careers. Philadelphia, PA: F. A. Davis.

[nhs12789-bib-0009] Braun, V. , & Clarke, V. (2006). Using thematic analysis in psychology. Qualitative Research in Psychology, 3(2), 77–101.

[nhs12789-bib-0010] Coupland, J. (2003). Small talk: Social functions. Research on Language & Social Interaction, 36(1), 1–6.

[nhs12789-bib-0011] De Meester, K. , Verspuy, M. , Monsieurs, K. G. , & Van Bogaert, P. (2013). SBAR improves nurse–physician communication and reduces unexpected death: A pre and post intervention study. Resuscitation, 84(9), 1192–1196. 23537699 10.1016/j.resuscitation.2013.03.016

[nhs12789-bib-0012] Deci, E. L. , Olafsen, A. H. , & Ryan, R. M. (2017). Self‐determination theory in work organizations: The state of a science. Annual Review of Organizational Psychology and Organizational Behavior, 4(1), 19–43.

[nhs12789-bib-0013] Edmondson, A. C. (2018). The fearless organization: Creating psychological safety in the workplace for learning, innovation, and growth. Hoboken, NJ: Wiley.

[nhs12789-bib-0014] Fisher, K. , Grosh, A. , & Felty, V. (2016). Using nurse‐led rounds to improve quality measures related to HAPUs. Nursing, 46(11), 63–68. 10.1097/01.NURSE.0000494657.67378.00 27759728

[nhs12789-bib-0015] Gagné, M. , & Deci, E. L. (2005). Self‐determination theory and work motivation. Journal of Organizational Behavior, 26(4), 331–362.

[nhs12789-bib-0016] Galletta, M. , Portoghese, I. , Carta, M. G. , D'Aloja, E. , & Campagna, M. (2016). The effect of nurse‐physician collaboration on job satisfaction, team commitment, and turnover intention in nurses. Research in Nursing & Health, 39(5), 375–385. 27233052 10.1002/nur.21733

[nhs12789-bib-0017] Garvey, M. I. , Bradley, C. W. , Wilkinson, M. A. C. , Holden, K. L. , Clewer, V. , & Holden, E. (2019). The value of the infection prevention and control nurse led MRSA ward round. Antimicrobial Resistance and Infection Control, 8(1), 53–55. 30911379 10.1186/s13756-019-0506-6 PMC6417022

[nhs12789-bib-0018] Hayes, L. J. , O'Brien‐Pallas, L. , Duffield, C. , Shamian, J. , Buchan, J. , Hughes, F. , … North, N. (2012). Nurse turnover: A literature review ‐ an update. International Journal of Nursing Studies, 49(7), 887–905. 22019402 10.1016/j.ijnurstu.2011.10.001

[nhs12789-bib-0019] Heaton, J. (2008). Secondary analysis of qualitative data: An overview. Historical Social Research / Historische Sozialforschung, 33(3), 33–45.

[nhs12789-bib-0020] Keyton, J. , Caputo, J. M. , Ford, E. A. , Fu, R. , Leibowitz, S. A. , Liu, T. , … Wu, C. (2013). Investigating verbal workplace communication behaviors. Journal of Business Communication, 50(2), 152–169.

[nhs12789-bib-0021] Levesque, C. , Zuehlke, A. N. , Stanek, L. R. , & Ryan, R. M. (2004). Autonomy and competence in German and American university students: A comparative study based on self‐determination theory. Journal of Educational Psychology, 96(1), 68–84.

[nhs12789-bib-0022] Noguchi‐Watanabe, M. , Yamamoto‐Mitani, N. , & Takai, Y. (2016). How does collegial support increase retention of registered nurses in homecare nursing agencies? A qualitative study. BMC Nursing, 15(1), 35. 27257406 10.1186/s12912-016-0157-3 PMC4890275

[nhs12789-bib-0023] Patton, M. Q. (2015). Qualitative research & evaluation methods: Integrating theory and practice (4th ed.). Thousand Oaks, CA: SAGE.

[nhs12789-bib-0024] Perreira, T. A. , Innis, J. , & Berta, W. (2016). Work motivation in health care: A scoping literature review. International Journal of Evidence‐Based Healthcare, 14(4), 175–182. 27552534 10.1097/XEB.0000000000000093

[nhs12789-bib-0025] Ruggiano, N. , & Perry, T. E. (2019). Conducting secondary analysis of qualitative data: Should we, can we, and how? Qualitative Social Work, 18(1), 81–97. 30906228 10.1177/1473325017700701 PMC6428200

[nhs12789-bib-0026] Ryan, R. M. , & Deci, E. L. (2000). Intrinsic and extrinsic motivations: Classic definitions and new directions. Contemporary Educational Psychology, 25(1), 54–67. 10620381 10.1006/ceps.1999.1020

[nhs12789-bib-0027] Ryan, R. M. , & Deci, E. L. (2017). Self‐determination theory: Basic psychological needs in motivation, development, and wellness. New York, NY: Guilford Press.

[nhs12789-bib-0028] Sasso, L. , Bagnasco, A. , Catania, G. , Zanini, M. , Aleo, G. , Watson, R. , & RN4CAST @IT Working Group . (2019). Push and pull factors of nurses' intention to leave. Journal of Nursing Management, 27(5), 946–954. 30614593 10.1111/jonm.12745

[nhs12789-bib-0029] Smeds Alenius, L. , Tishelman, C. , Runesdotter, S. , & Lindqvist, R. (2014). Staffing and resource adequacy strongly related to RNs' assessment of patient safety: A national study of RNs working in acute‐care hospitals in Sweden. BMJ Quality & Safety, 23(3), 242–249. 10.1136/bmjqs-2012-001734 PMC3932760 24125740

[nhs12789-bib-0030] Terry, G. , Hayfield, N. , Clarke, V. , & Braun, V. (2017). Thematic analysis In Willig C. & Stainton Rogers W. (Eds.), The SAGE handbook of qualitative research in psychology (2nd ed., pp. 17–37). Thousand Oaks, CA: Sage.

[nhs12789-bib-0031] Toode, K. , Routasalo, P. , & Suominen, T. (2011). Work motivation of nurses: A literature review. International Journal of Nursing Studies, 48(2), 246–257. 20947085 10.1016/j.ijnurstu.2010.09.013

[nhs12789-bib-0032] van den Broeck, A. , Ferris, D. L. , Chang, C.‐H. , & Rosen, C. C. (2016). A review of self‐determination theory's basic psychological needs at work. Journal of Management, 42(5), 1195–1229.

[nhs12789-bib-0033] Wanzer, M. B. , Wojtaszczyk, A. M. , & Kelly, J. (2009). Nurses' perceptions of physicians' communication: The relationship among communication practices, satisfaction, and collaboration. Health Communication, 24(8), 683–691. 20183377 10.1080/10410230903263990

[nhs12789-bib-0034] Weller, J. , Boyd, M. , & Cumin, D. (2014). Teams, tribes and patient safety: Overcoming barriers to effective teamwork in healthcare. Postgraduate Medical Journal, 90(1061), 149–154. 24398594 10.1136/postgradmedj-2012-131168

[nhs12789-bib-0035] World Health Organization . (2016). Global strategy on human resources for health: Workforce 2030. Geneva, Switzerland: World Health Organization.

[nhs12789-bib-0036] World Medical Association . (2013). World Medical Association Declaration of Helsinki: Ethical principles for medical research involving human subjects. JAMA, 310(20), 2191–2194. 24141714 10.1001/jama.2013.281053

[nhs12789-bib-0037] Ylitörmänen, T. , Turunen, H. , Mikkonen, S. , & Kvist, T. (2019). Good nurse–nurse collaboration implies high job satisfaction: A structural equation modelling approach. Nursing Open, 6(3), 998–1005. 31367424 10.1002/nop2.279 PMC6650654

